# Gut microbiota *Parabacteroides distasonis* enchances the efficacy of immunotherapy for bladder cancer by activating anti-tumor immune responses

**DOI:** 10.1186/s12866-024-03372-8

**Published:** 2024-07-03

**Authors:** Benlin Wang, Yifeng Qiu, Ming Xie, Pengcheng Huang, Yao Yu, Qi Sun, Wentai Shangguan, Weijia Li, Zhangrui Zhu, Jingwen Xue, Zhengyuan Feng, Yuexuan Zhu, Qishen Yang, Peng Wu

**Affiliations:** Department of Urology, Nanfang Hospital, Southern Medical University, Guangzhou, China

**Keywords:** Gut microbiota, Bladder cancer, *Parabacteroides distasonis*, Immune checkpoint inhibitors

## Abstract

**Objective:**

Bladder cancer(BCa) was a disease that seriously affects patients’ quality of life and prognosis. To address this issue, many researches suggested that the gut microbiota modulated tumor response to treatment; however, this had not been well-characterized in bladder cancer. In this study, our objective was to determine whether the diversity and composition of the gut microbiota or the density of specific bacterial genera influence the prognosis of patients with bladder cancer.

**Methods:**

We collected fecal samples from a total of 50 bladder cancer patients and 22 matched non-cancer individuals for 16S rDNA sequencing to investigate the distribution of *Parabacteroides* in these two groups. Further we conducted follow-up with cancer patients to access the impact of different genera of microorganisms on patients survival. We conducted a Fecal Microbiota Transplantation (FMT) and mono-colonization experiment with *Parabacteroides distasonis* to explore its potential enhancement of the efficacy of anti-PD-1 immunotherapy in MB49 tumor-bearing mice. Immunohistochemistry, transcriptomics and molecular experiment analyses were employed to uncover the underlying mechanisms.

**Results:**

The 16S rDNA showed that abundance of the genus *Parabacteroides* was elevated in the non-cancer control group compared to bladder cancer group. The results of tumor growth curves showed that a combination therapy of *P. distasonis* and ICIs treatment significantly delayed tumor growth and increased the intratumoral densities of

both CD4^+^T and CD8^+^T cells. The results of transcriptome analysis demonstrated that the pathways associated with antitumoral immune response were remarkably upregulated in the *P. distasonis* gavage group.

**Conclusion:**

*P. distasonis* delivery combined with α-PD-1 mAb could be a new strategy to enhance the effect of anti-PD-1 immunotherapy. This effect might be achieved by activating immune and antitumor related pathways.

**Supplementary Information:**

The online version contains supplementary material available at 10.1186/s12866-024-03372-8.

## Introduction

Immune checkpoint inhibitors (ICIs) had demonstrated potent antitumor activity against various types of cancers. Anti-programmed cell death 1/programmed cell death ligand 1(PD-1/PD-L1) inhibitors were recommended in clinical guidelines as first and second-line treatments for select patients with advanced bladder cancer [1]. However, their long-term efficacy had been observed in a small subset of patients, with most unable to benefit from ICIs due to treatment resistance, encompassing both primary and acquired resistance [2, 3]. The prediction and enhancement of drug resistance were pivotal factors determining the effectiveness of anti-PD-1/PD-L1 therapies.

The microbiome was defined as the collective genomes of microbes within a community,whereas the term microbiota refered to the microbes themselves in aggregate. Within a human organism, there are trillions of microbes which interact with the host constantly at numerous sites [4]. Several trillions of microbes exit in human organism. During the human development, gut microbiota constantly interacted with the host in many parts of the body and exert a variety of extremely important role in host function, especially immunity [5]. Therefore, an increasing body of research evidence suggested that the gut microbiota influenced the efficacy of immunotherapy by affecting host immunity [6, 7]. However, the dominant microbiota impacting responses might vary across different tumor types and immunotherapies [8]. According to the reports, melanoma patient with a high relative abundance of favorable microbiota, such as *Clostridiales*, *Ruminoccaceae*, and *Faecalibacterium*, increased antigen presentation and improved effector CD4^+^T cells and CD8^+^T cell function in the perpheral blood and TME to ameliorate the antitomor efficacy of ICIs [9]. Besides, numerous other bacterial strains had been shown to enhance the efficacy of PD-1 inhibitors through diverse mechanisms, including *Bifidobacterium bifidum* [6, 10], *Enterococcus faecium* [11], *Lactobacillus rhamnosus GG* [12], and *Ackermann bacteria* [13]. These gut microbiota all enhanced the efficacy of immunotherapy by mediating specific signaling pathways to increase the infiltration of immune cells.

Furthermore, *Parabacteroides distasonis* played an important role in human health as a probiotic. It improved non-tumoral diseases such as liver fibrosis,inflammatory arthritis and obesity by affecting bile acid metabolism [14–16]. It also been reported to influence PD-1/PD-L1 immunotherapy outcomes in solid tumors. In a melanoma transplant model, an increased abundance of *P. distasonis* correlated with a heightened response to a combined anti-CTLA-4 and anti-PD-1 antibody treatment [17]. Similarly, in the context of bladder cancer, probiotic probio-M9 treatment in mice significantly enhanced their response to immunotherapy and led to a notable increase in *P. distasonis* abundance [18]. Experimental evidence had shown significantly higher levels of *P. distasonis* in the feces of patients in the immunotherapy response group. Moreover, ginseng polysaccharide treatment in mice enhanced their immunotherapy response and elevated *P. distasonis* abundance [19].

This study aims to investigate the prevalence of *P. distasonis* in bladder cancer patients and healthy controls using 16S rDNA gene sequencing. It also seek to confirm its impact and the associated mechanism on the anti-PD-1/PD-L1 immunotherapy in bladder cancer, employing an animal model of FMT and mono-colonization with *P. distasonis*. The findings from this research will serve as a reference for predicting and enhancing the efficacy of ICIs in patients with bladder cancer.

## Material and methods

### Patient cohort and sample collection

Samples from BCa patients were validated through postoperative pathology, by postoperative pathology, confirming that the paraffin samples all represented urothelial carcinomas. Additionally, cases of in situ carcinoma were meticulously excluded following examination by two independent pathologists, who also verified that the samples had not undergone any antitumor treatment. The exclusion criteria for both BCa patients and non-cancer individuals were as follows: 1) Usage of drugs known to influence the gut microbiota within one month prior collection of fecal samples, including antibiotics and proton pump inhibitors and 2) Suffering gastrointestinal diseases that could potentially cause dysbiosis of the gut microbiota, such as diarrhea, constipation, or inflammatory bowel disease (IBD). A total of 50 bladder cancer patients were included in our present study, among which 39 patients were non-muscle invasive bladder cancer (NMIBC) and other 11 patients were muscle invasive bladder cancer (MIBC) according to post-operation pathological diagnosis. Because recurrence being a primary clinical outcome in NMIBC cases, we performed a postoperative follow-up on the 39 NMIBC patients within our clinical cohort and categoried them into two groups: the recurrence group (R group) and the non-recurrence group (NR group), based on whether they experienced disease recurrence or not. The fecal samples were collected from both BCa patients and controls during natural defecation using sterile fecal sample collectors and were promptly stored at -80℃ within 2 hours.

### DNA extraction and amplification

We extracted total DNA from the fecal samples obtained from both BCa patients and individuals without cancer using the Omega Mag-Bind soil DNA kit (Omega Bio-Tek, Norcross, GA, USA). To assess the quality of the isolated DNA, we performed 1% agarose gel electrophoresis. The V4 region of 16S rDNA of bacteria was amplified. The specific primer sequences for V4 region were 5’-GTGTGYCAGCMGCCGCGGTAA-3’ (Forward primer) and 5’-CCGGACTACNVGGGTWTCTAAT-3’ (Reverse primer). Subsequently, we constructed the 16S rDNA sequencing library using NEBNext® Ultra™ IIDNA Library Prep Kit (Cat No. E7645) and paired-end sequencing was performed on an Illumina NovaSeq platform.

### 16S rDNA analyses and differential flora analysis

Denoising and filtering were carried out using the DADA2 method within in the QIIME2 software. Subsequently, amplicon sequence variant (ASV) was obtained [20]. Species annotation was achieved in QIIME2 using the SILVA database and the naive Bayes classifier [21]. The α-diversity of the gut microbiota was assessed by calculating four distinct indices in QIIME2, which include Observed_otus, Shannon index, Simpson index, and Pielou_e index. Bacterial community richness was evaluated using Observed_otus, while species evenness was assessed using Pielou’s evenness index. Bacterial community diversity was evaluated using the Shannon index and Simpson index. To compare the differences in diversity indices between two groups, the Wilcoxon test was employed. Furthermore, the β-diversity of the gut microbiota was analyzed by calculating both the Bray-Curtis distance and weighted UniFrac distance in QIIME2 [22].

Principal-coordinate analysis (PCoA) and non-metric multidimensional scaling (NMDS) analyses were employed to visualize and represent the dissimilarities in microbial composition between the two groups, utilizing the Bray-Curtis and weighted UniFrac distance matrices. To assess the variation in β-diversity between BCa and control groups, Permutational multivariate analysis of variance (PERMANOVA) was conducted.

The “circlize” package was utilized to visualize the relative abundance of major bacteria at phylum, class, order, family and genus levels in both BC and control groups. The “survminer” package was used to differentiate NMIBC patients through the optimal cut-off value of bacteria relative abundance. This approach can reveal a more pronounced difference between the two groups in Kaplan-Meier analysis.We examined the difference in the abundance of bacterial genera between the two groups and identified differential genera on the Microbiota-Analyst online platform [23]. A volcano plot was constructed to depict the differential genera meeting the criteria of |log_2_FC| >1 and FDR < 0.05. Specific bacterial identification was performed using the Linear Discriminant Analysis (LDA) Effect Size (LEfSe) method. The Wilcoxon test was used to compare the relative abundance of bacteria between the two distinct groups.

### Cell culture

The murine bladder cancer cell MB49 were cultured in DMEM(Gibco) supplemented with 10% fetal bovine serum(Gibco) and 100 U/mL penicillin/streptomycin(China) in a humidified incubator, which contains 5% CO_2_ at 37°C. MB49 cells were harvested during their logarithmic growth phase for subcutaneous tumor experiments.

### Bacterial strains culture

*P. distasonis*, which was acquired from the American Type Culture Collection(ATCC, USA), was cultured in BHI broth as required. Under anoxic condition in a shaking incubator at 37°C with 10% H_2_, 80% N_2_, and 10% CO_2_, it was subsequently resuspended in BHI broth before administration by gavage.

### Animal experiments

Female 5–7 weeks old C57BL/6J mice were evenly divided into five groups: BC + α-PD-1 mAb group: Mice received FMT from bladder cancer patients along with α-PD-1 monoclonal antibody (mAb) at a dose of 250 µg per mouse (clone: RMP1-14, Bio X Cell) ; N+ α-PD-1 mAb group: Mice that received FMT from healthy individuals along with α-PD-1 mAb; Pa + α-PD-1 mAb group: Mice that were mono-colonized with P. distasonis and received α-PD-1 mAb; mAb group: Mice treated with a vehicle (PBS) along with α-PD-1 mAb and control group (*n*=6). To establish a transplanted tumor model, approximately 2×10^6^ MB49 cells were inoculated into the left flanks of the mice. When the tumors reached a size of 100 mm^3^, PD-1 drug therapy was initiated. Tumor volume was monitored every three days with vernier calipers and calculated using the formula: length×width^2^×0.52.

### Fecal microbiota transfer and *bacteria*-colonized experiment

Based on the method previously described with modifications, the FMT experiment was conducted [24]. Specifically, fecal samples from the donors BCa patients and healthy individuals were aseptically collected into centrifuge tubes and then resuspended in PBS at a concentration of 125 mg/mL. Following this, the mixtures underwent centrifugation at 1000×g for 1 minute. The resulting supernatants were then carefully transferred and stored in separate 1.5 mL tubes, prepared for future microbiota transplantation. Before FMT experiment, the acceptor C57BL/6 mice were given antibiotics, which included ampicillin (1 g/L, Aladdin), neomycin sulfate(1 g/L, Aladdin), metronidazole (1 g/L, Aladdin), and vancomycin (0.5 g/L, Macklin) intragastrically once a day for one week to deplete the gut microbiota. Next, the Pa + mAb group received a daily gavage of 200µL containing 2.0 CFU of *P. distasonis* for a duration of 2 weeks. The N + mAb group and BC + mAb group were administered a daily gavage of 200µL containing a 3.0 CFU suspension. The mAb group and the control group received an equivalent volume of PBS by gavage.

### Immunohistochemistry

Immunohistochemistry (IHC) was performed on sections of bladder cancer tissues that had been fixed in 10% formalin and embedded in paraffin. All sections were counterstained with hematoxylin, dehydrated, mounted, and processed using peroxidase-conjugated avidin/biotin and 3’-3-diaminobenzidine (DAB). The primary antibodies used in this study included monoclonal mouse anti-CD4^+^T (Abcam, 1:2000), monoclonal mouse anti-CD8^+^T (Abcam, 1:4000), and monoclonal mouse anti-FoxP3^+^ (Abcam, 1:200). Two independent pathologists assessed all sections in a blind fashion. The molecular expressions of CD4^+^T, CD8^+^T, and FoxP3^+^ were determined by calculating the ratio of positive staining cells to total tumor cells. The density of CD4^+^ T, CD8^+^ T, FoxP3^+^ cells was quantified as the number of positive cells per square millimeter (cells/mm^2^) and was counted in five random areas (1 mm^2^ /each) , with the average of the five fields taken into account.

### RNA sequencing and analysis

Firstly, we extracted total RNA from the subcutaneous tumors and determined the RNA concentration using a NanoDrop2000 Spectrophotometer. Subsequently, we employed the KAPA RNA library prep kit to construct RNA sequencing libraries following the manufacturer’s instructions. The Illumina NovaSeq platform was used for RNA sequencing. The reference genome (GRCm39) for mice was downloaded from the GENCODE database (https://www.gencodegenes.org/mouse/).

We used the R package “edgeR” to identify differentially expressed genes(DEGs) between the Pa + mAb group (*n*=2) and BC +mAb group(*n*=2). Genes with |log_2_FC| >2 and *p*-value < 0.05 were considered as DEGs. The Kyoto Encyclopedia of Genes and Genomes (KEGG) enrichment analysis of DEGs was conducted using the “ClusterProfiler” package, and the results were visualized using the “enrichplot” package. Additionally, we employed Gene Set Enrichment Analysis (GSEA) to explore the difference in functional pathways between the Pa + mAb group and the BC + mAb group, the KEGG gene sets for GSEA analysis were obtained through the "msigdbr" package (version 7.5.1) [25].

### Statical analysis

Utilizing GraphPad Prism 8.0 for statistical analyses, variables were expressed as Means ± SEM, as elaborated in the figure legends. For comparisons among three or more groups, one-way ANOVA and Tukey’s tests were used. The significance threshold was set at *p*<0.05. Significance levels were denoted as follows: *p<0.05, **p<0.01, ***p<0.001, ****p<0.0001, ns indicates no significant difference.

## Results

### The clinical characteristics of the study cohort and the impact of disease status on the fecal microbiota

**Fig. 1:**
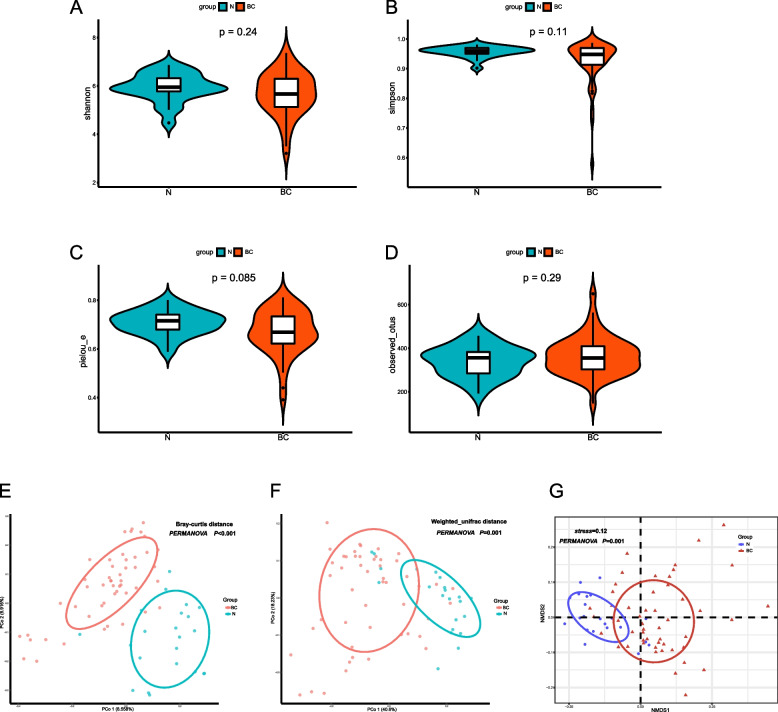
16S rDNA profiling of the gut microbiota between BC group and control group. The α-diversity of Shannon index (**A**); Simpson index (**B**); Pielou_e index (**C**); observed_otus (**D**) results showed no difference between the two groups. The PCoA based on Bray-curtis distance and Weighted UniFrac distance revealed a marked difference in microbial composition between the β-diversity of two groups. Non-metric multidimensional scaling (NMDS) analysis based on weighted UniFrac distance also showed a marked difference in microbial composition between the two groups (**E**)

In a study conducted at Nanfang Hospital from July 2018 to October 2021, we recruited 50 BCa patients (BC group) and 22 healthy individuals (control group) carefully matched based on exclusion criteria. Analysis of α-diversity revealed that there were no significant differences between the BC and control groups in the Shannon index (*p=*0.24), Simpson Index (*p=*0.11), and Observed_otus(*p*=0.29)(Fig. 1A,B,D). A slightly higher Pielou_e index of gut microbiota was observed in the control group compared to the BC group, the difference was not statistically significant(*p*=0.085) (Fig. 1C). Further analyses using PCoA and PERMANOVA demonatrated notable distinctions in microbial compositions between BC group and control group, based on Bray-cutis distance (*p*<0.001) and Weighted UniFrac distance(*p*=0.001). NMDS analysis also highlighted significant differences in gut microbiota composition between the two groups (*p*=0.001)(Fig. 1E).

**Fig. 2:**
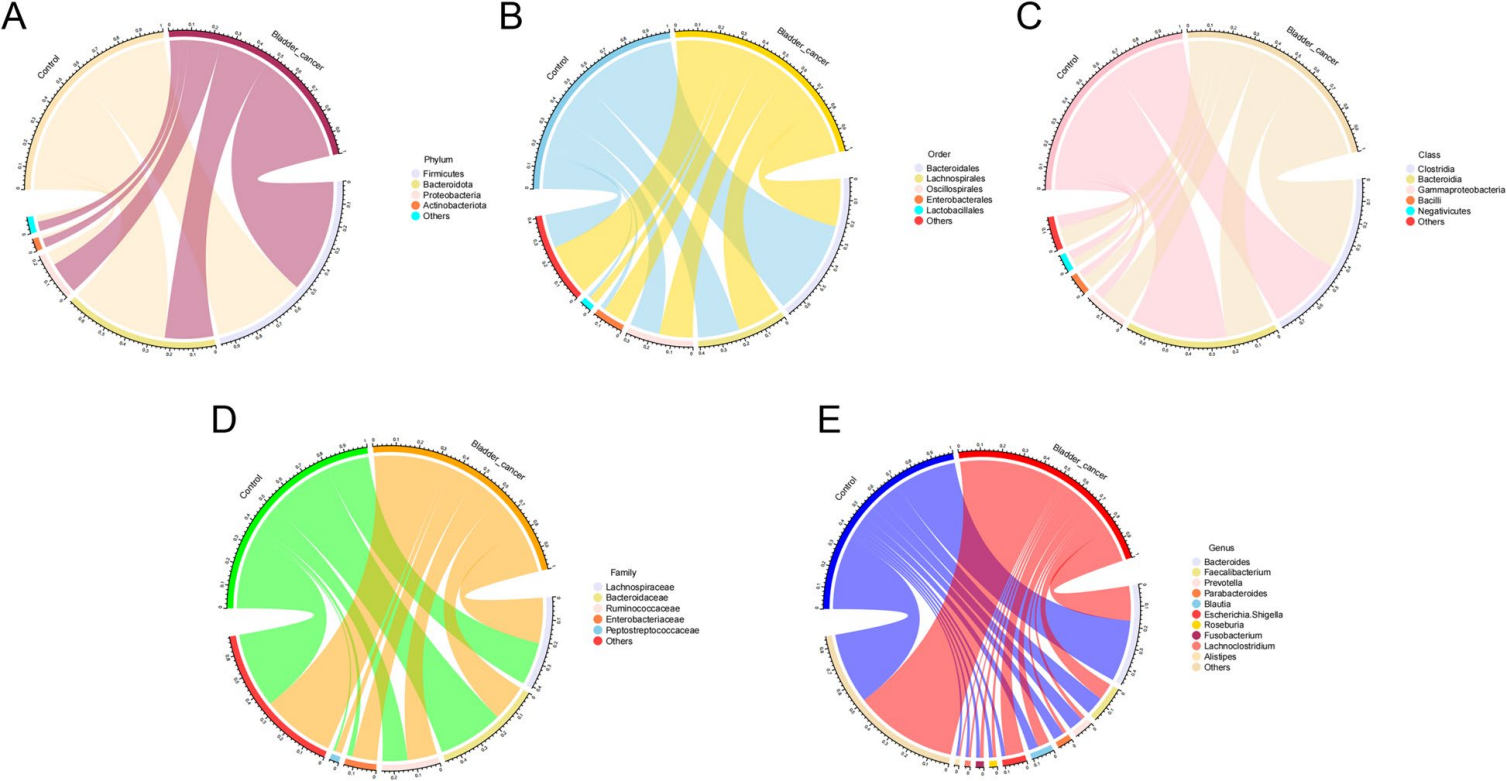
Chord diagram display of the relative abundance of the major taxa. This chord diagram represents the relative abundance of major taxonomic groups within the BC and control groups across five taxonomic levels: Phylum (**A**), Class (**B**), Order (**C**), Family (**D**) and Genus (**E**)

**Fig. 3:**
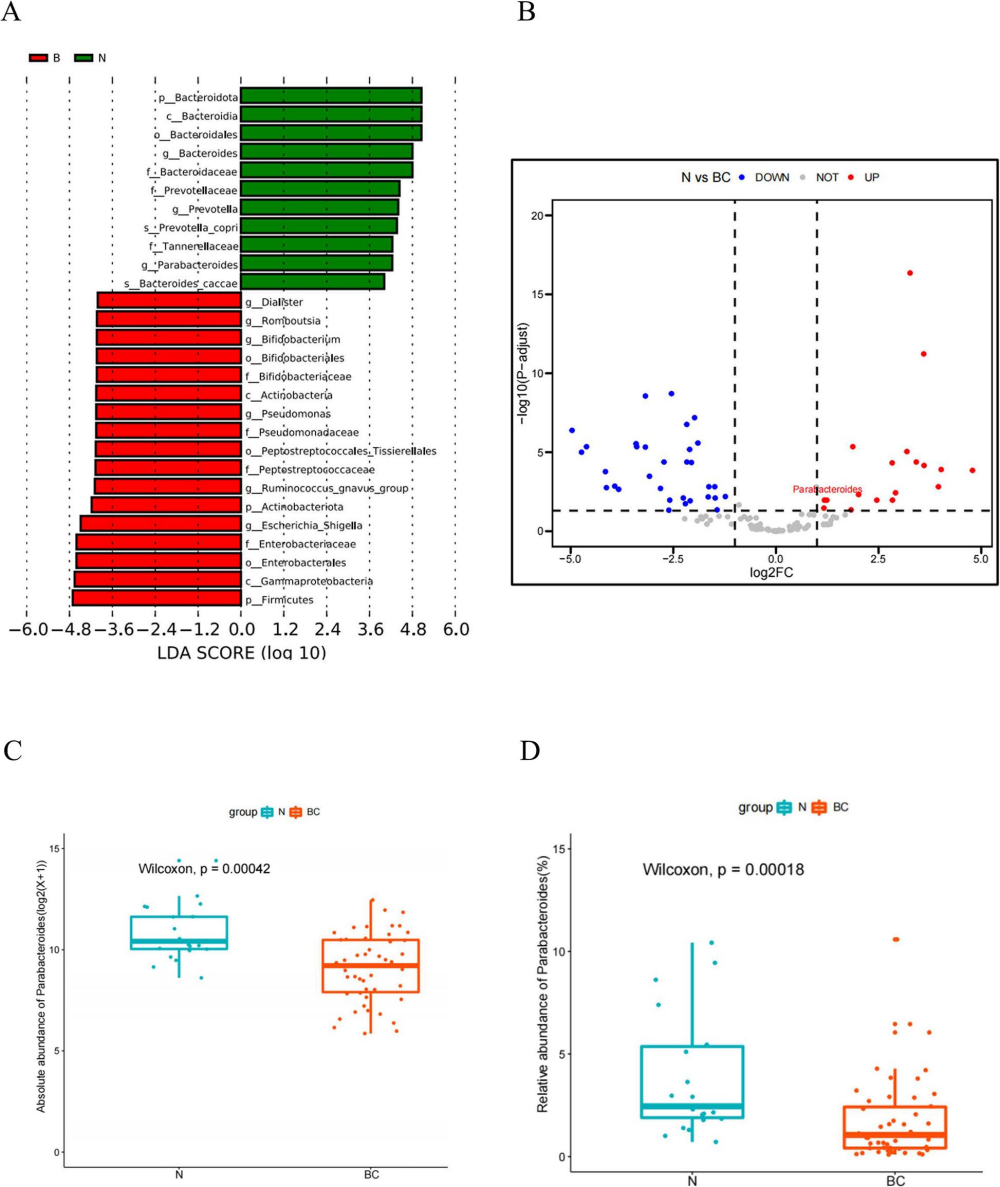
Identification of differential abundant taxa between the BC group and the control group. Results and LDA score bar plot of the LEfSe analysis (**A**); Volcano plot displayed the differential abundant genera between the two groups (**B**); Marked differences in the absolute abundance (**C**) and the relative abundance (**D**) of the genus Parabacteroides between the two groups

The chord diagram represented the relative abundance of major taxonomic groups within the BC and control groups across five taxonomic levels. Each level aimed to reveal the relationships and relative proportions between different biological taxa.At the phylum level, four major taxa were observed: *Firmicutes*, *Bacteroidota*, *Proteobacteria*, and *Actinobacteriota* (Fig. 2A) ,and the major taxa at the Family, Order, and Class levels also were displayed (Fig. 2B-D). The ten most abundant taxa at the genus level were shown, including *Bacteroides*, *Faecalibacterium*, *Prevotella*, *Parabacteroides*, *Blautia*, *Escherichia-Shigella*, *Roseburia*, *Fusobacterium*, *Lachnoclostridium*, and *Alistipes* (Fig. 2E). The diagram intuitively displayed the interactions and connections between different taxa through linking lines, providing deep insights into the variations in microbial diversity under different conditions or environments. Furthermore, through LEfSe analysis comparing the differences in the distribution of the microbial communities at the genus level between the two groups, it was found that *Bacteroides*, *Parabacteroides* and *Prevotellaceae* were more abundant in the control group. Meanwhile, the BC group exhibited a higher relative abundance of *Romboutsia*, *Bifidobacterium*, and *Pseudomonas* at the genus level (Fig. 3A).We identified 50 genera with significantly different abundance between the two groups using the DESeq2 method. Among these, 18 genera showed a significant increasing in the control group, including *Parabacteroides* (Fig. 3B). Both the absolute abundance(*p*<0.001) and relative abundance (*p*<0.001) of the genus *Parabacteroides* were significantly in the control control group compared with those in the BC group(Fig. 3C and D).

**Fig. 4:**
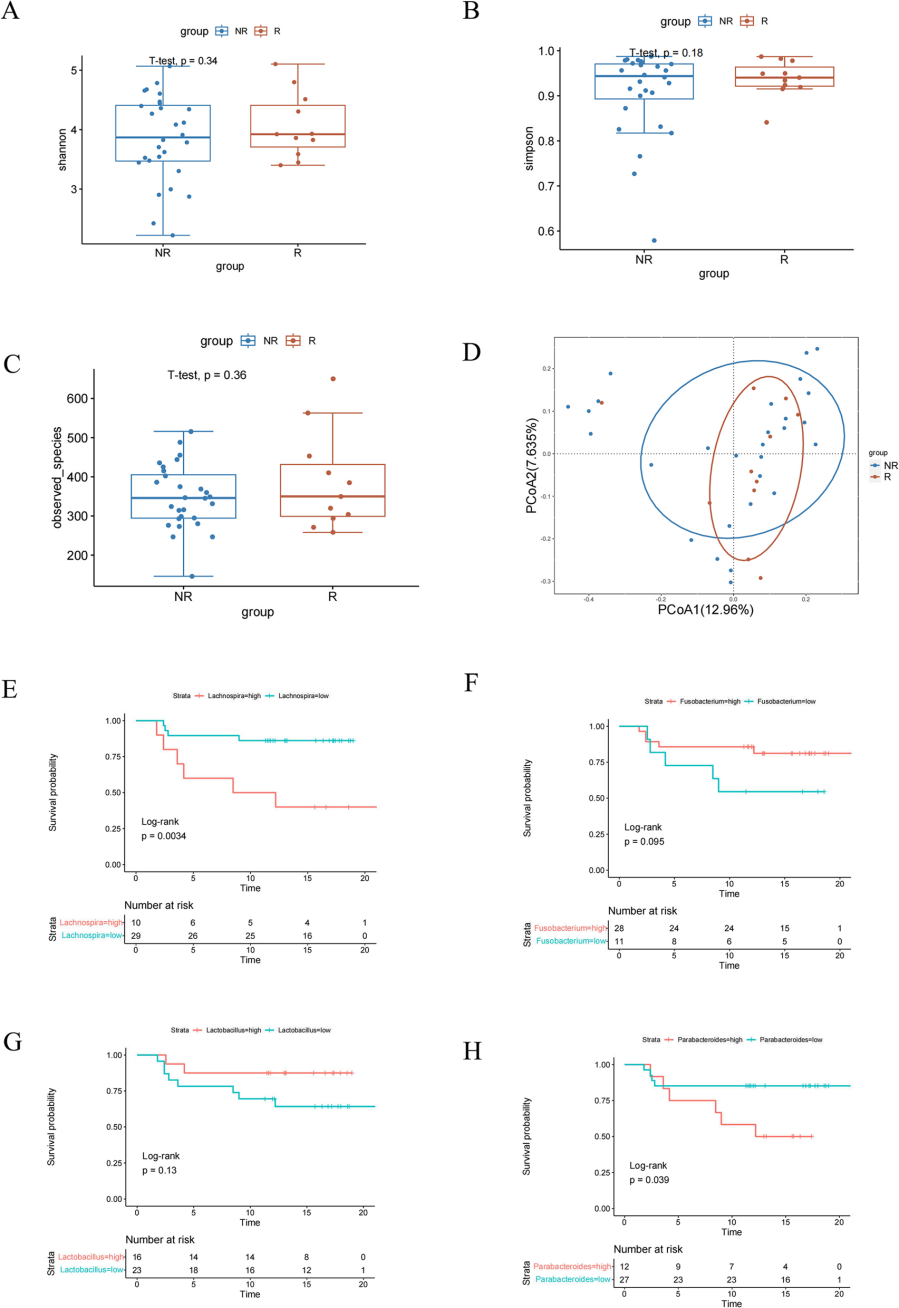
16S rDNA sequencing analysis and Kaplan-Meier progression-free survival curves disclosed the association between patient prognosis and gut microbiota in both R and NR group. The α-diversity of Shannon index (**A**); Simpson index (**B**) and Observed species (**C**) results showed no difference between the two groups. The β-diversity of PCoA result significantly differentiated between the R group and the NR group (**D**). Kaplan-Meier survival analysis according to the abundance of *Lachnospira* (**E**), *Fusobacterium* (**F**), *Lactobacillus* (**G**) and *Parabacteroides* (**H**)

To investigate the potential associations between gender or smoking habits and the gut microbiome in bladder cancer patients, we assessed the microbiome composition differences across genders and smoking statuses. Our analysis revealed no significant disparities in the Shannon index(*p*=0.34), Simpson Index(*p*=0.73), and Observed_species (*p*=0.055) among male and female bladder cancer patients (Supplement Figure 1A-C). Similarly, no significant differences in Shannon index(*p*=0.089), Simpson Index(*p*=0.22), and Observed_species(*p*=0.58) were observed in gut microbiota α-diversity between patients with smoking habit versus not(Supplement Figure 1E-G). Results from PCoA analysis further confirmed that the composition of the gut microbiota was comparable between males and females(*p*=0.06), as well as between smokers and non-smokers (*p*=0.56) (Supplement Figure 1D and H). Therefore, we considered that the differences in gut microbiota composition could be related to the disease status of the patients. We proceeded to conduct follow-up statistics on 39 NMIBC patients and categorized them into two groups: the recurrence group (R group, *n*=11) and the non-recurrence group(NR group, *n*=28). The detailed baseline characteristics of NMIBC patients were presented in Table 1. Subsequently, we distinguished NMIBC patients based on the optimal cut-off value of bacteria relative abundance by using the "surv_cutpoint" function from the "survminer" package, This approach revealed a more pronounced difference between the two groups in Kaplan-Meier analysis. The results of α-diversity analysis showed that there were no significant differences between the R and NR groups in the Shannon index (*p*=0.34), Simpson Index (*p*=0.18) and Observed_species (*p*=0.36)(Fig. 4A-C). Results from PCoA indicated that R and NR group had notably distinct microbial compositions(Fig. 4D). The results of the survival curve analysis uncovered a significant relationship between the abundance of specific bacteria and patient prognosis. Specifically, an increase in the abundance of *Lachnospira* was associated with an extended prognosis in patients (*p*=0.0034) (Fig. 4E). Furthermore, while no statistical difference was observed in the abundance of *Fusobacterium* (*p*=0.095) and *Lactobacillus*(*p*=0.13) (Fig. 4F and G). However, it was worth mentioning that patients with a higher relative abundance of the Parabacteroides genus had significantly worse recurrence-free survival compared to those with lower abundance(*p*=0.039)(Fig. 4H). This could be attributed to our use of 16s rDNA sequencing to analyze the gut microbiota of bladder cancer patients, which only measured abundance at the genus level. The paradoxical results suggested that different species of *Parabacteroides* might have distinct effects on bladder cancer. Although *Parabacteroides* genus did not effectively differentiate patient prognosis, this did not hinder our continued investigation into the role of the *Parabacteroides* species in immunotherapy for bladder cancer animal models.

**Table 1:** The detailed baseline characteristics of NMIBC patients

Characteristics		Patients (*N*=39)	R (*n*=11)	NR (*n*=28)	*P*
Ages(years)	Median(range)	59(29-86)	65(49-86)	57(29-86)	0.733
Sex	Male	32(82.05%)	10(90.91%)	22(78.57%)	0.635
	Female	7(17.95%)	1(9.09%)	6(21.43%)	
Smoking history	Yes	16(41.03%)	3(27.27%)	13(46.43%)	0.291
	No	23(8.97%)	8(72.72%)	15(53.57%)	
BMI	<24	22(56.41%)	6(54.55%)	16(57.14%)	0.747
	24-28	14(35.90%)	3(27.27%)	11(39.29%)	
	>28	3(7.69%)	2(18.18%)	1(3.57%)	
Grade	PUNMLP	11 (28.21%)	2(18.18%)	9(32.14%)	0.468
	Low	17(43.59%)	6(54.54%)	11(39.29%)	
	High	11(28.21%)	3(27.27%)	8(28.57%)

### *P.distasonis* enhances the effectiveness of anti-PD-1 treatment by increasing immune cell infiltration

**Fig. 5:**
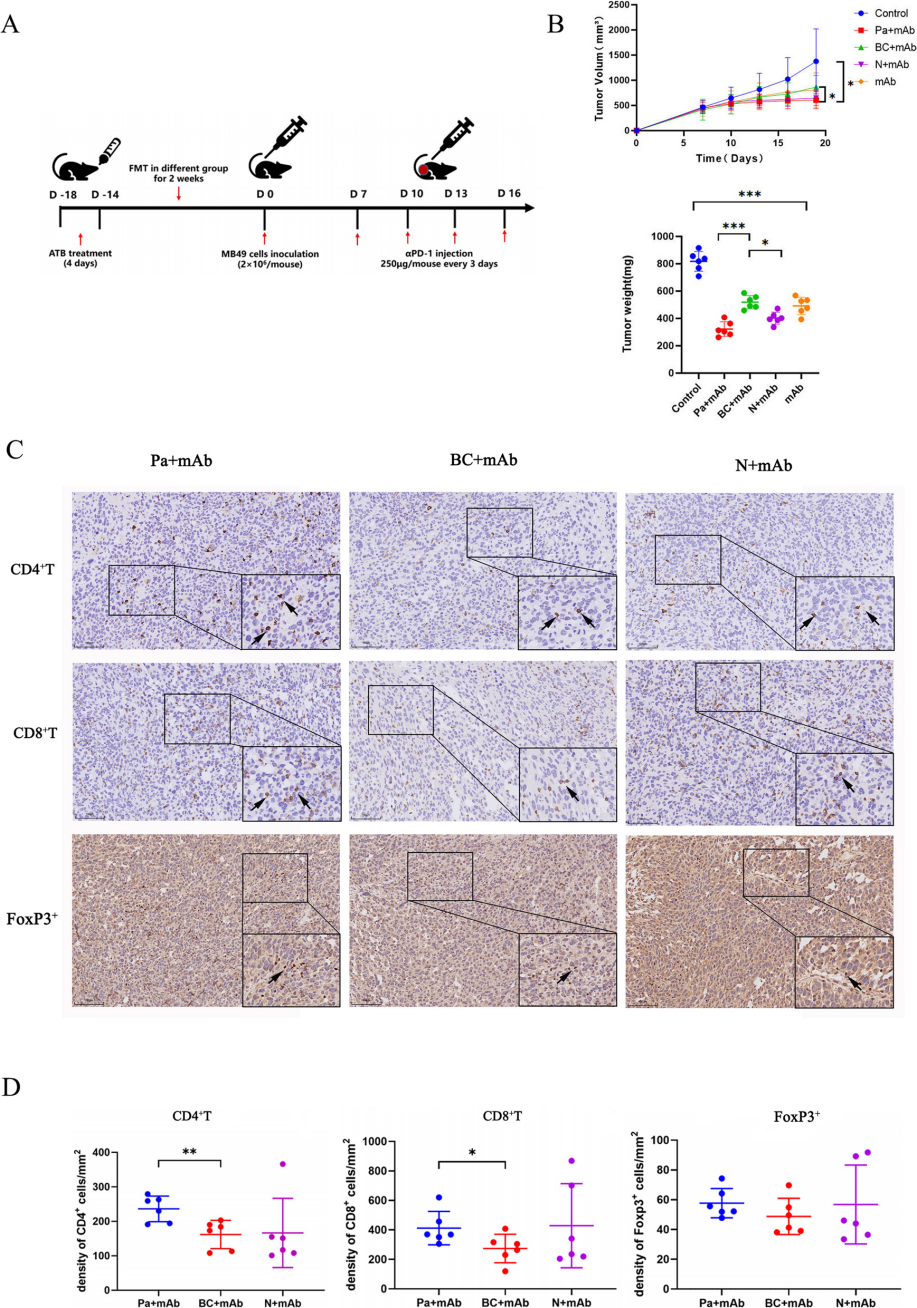
Gut microbial *P. distasonis* enhanced the α-PD-1 mAb efficacy through increasing immune cell infiltration. The Schematic diagram showing experimental design method (**A**); The images of tumor growth curve and tumor weight (**B**); Representative IHC profiles of CD4^+^T, CD8^+^T, FoxP3^+^ in tumor tissues at ×40 magnification. scale bar=100 µm (**C**); The graph of statistical analysis for the expression of CD4^+^T, CD8^+^ T cells and FoxP3^+^ in tumor tissues (**D**). Significant differences were evaluated by one-way ANOVA(* *p* < 0.05,** *p*< 0.01). Data were presented as Mean ± SEM

According to the LEfSe analysis and literature reports, we discovered that *Parabacteroides distasonis*, recognized as a beneficial bacterium, was being extensively studied. It played a crucial role in improving metabolism and inhibiting disease progression in non-tumor diseases [14, 16]. However, current research on its role in bladder cancer immunotherapy was lacking. Therefore, to explore the correlation between the two, we conducted an experiment to investigate whether *P. distasonis* could enhance the effectiveness of α-PD-1 mAb in MB49 tumor-bearing mice (Fig. 5A).

It has been reported that the gut microbiota in the host body could be transferred through FMT, resulting in the recipient exhibiting the same effects as the donor. Therefore, to more fully evaluate the enhancement of immunotherapy effects by *P. distasonis*, we conducted FMT with fecal samples from both BCa cancer patients and healthy control groups into mice. We then assessed the therapeutic effects by observing tumor growth differences between the groups. By regularly monitoring tumor volume growth and analyzing the tumor growth curves and tumor weight,we found that gavage with *P. distasonis* combined with α-PD-1 mAb treatment could significantly inhibit tumor growth and reduce tumor weight compared to the BC + α-PD-1 mAb treatment group (*p*<0.05, *p*<0.001) (Fig. 5B). The immune cell profile of MB49 bladder tumors was analyzed using immunohistochemistry. Data revealed that following mono-colonization by *P. distasonis* combined with the α-PD-1 mAb treatment significantly boosted the intratumoral densities of both CD4^+^T and CD8^+^ T cells, compared to the BC + α-PD-1 mAb treatment group(*p*<0.01, *p*<0.05). However, the density of intratumoral FoxP3^+^ cells showed no significant difference (Fig. 5C-D). This suggested that the antitumor effect in tumor-bearing mouse models may be enhanced by the combination of *P. distasonis* and α-PD-1 mAb through the promotion of increased immune cell infiltration.

### RNA-sequencing results indicated *P.distasonis* improved anti-PD-1 treatment efficacy through enhancing anti-tumor immune response

**Fig. 6:**
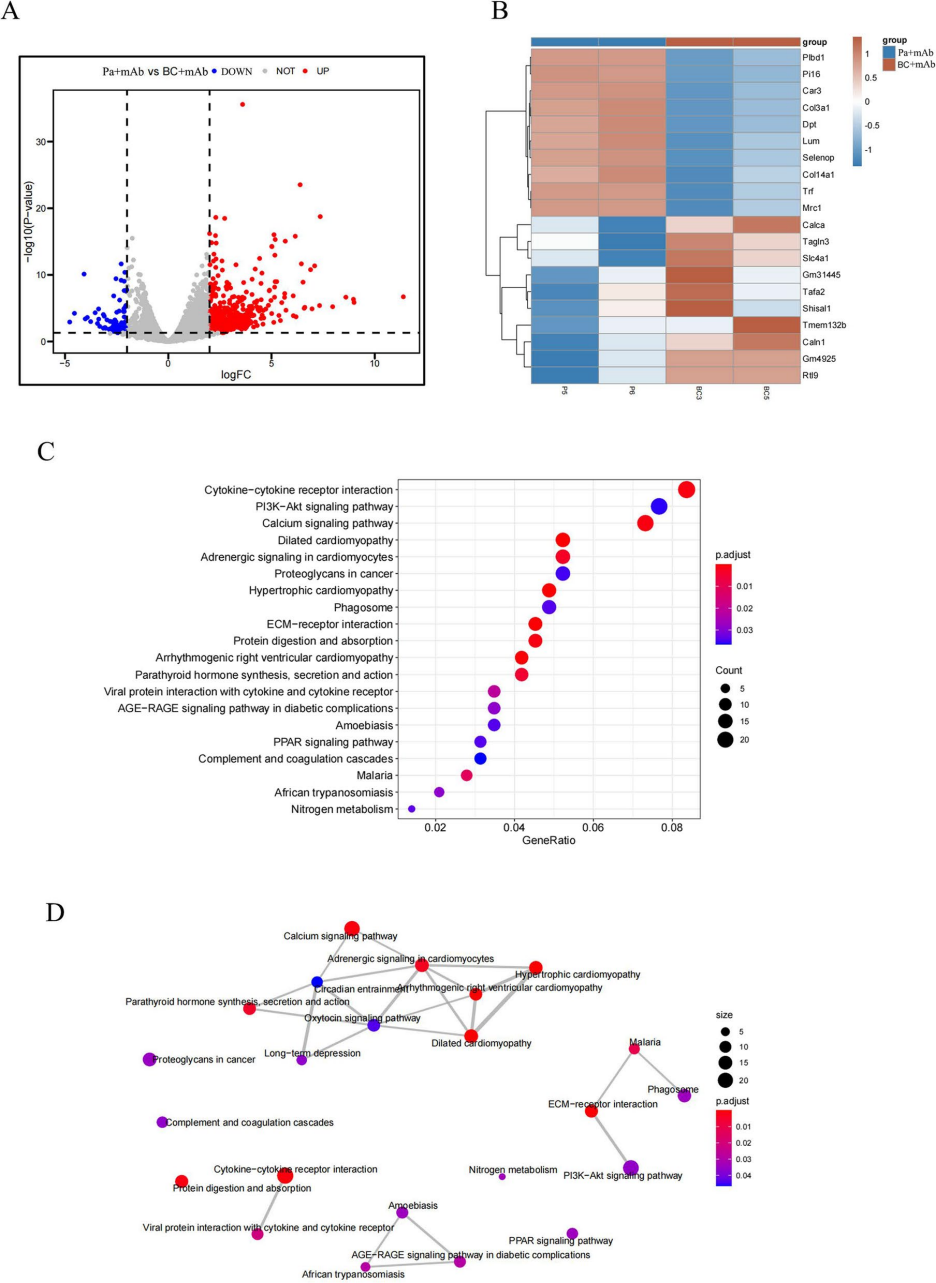
The results of RNA sequencing analysis revealed differential gene expression and pathway enrichment. The volcano plot showed the differentially expressed genes upregulated and downregulated between the two groups (**A**); The heatmap showed the top ten genes up in the Pa + mAb group and BC+mAb group (**B**); The bubble plot (**C**) and network analysis (**D**) revealed that these DEGs were closely associated with some tumor-related pathways

To elucidate the molecular mechanisms underlying the enhancement of anti-PD-1 treatment efficacy by *P.distasonis*, we performed RNA sequencing of tumor tissue collected from Pa + mAb group(*n*=2) and BC + mAb group(*n*=2). According to the transcriptome analysis volcano plot, we screened 699 DEGs between BC + mAb and Pa + mAb groups, among which 600 genes were markedly upregulated and 99 genes were downregulated in the Pa + mAb group compared with those in the BC+mAb group (Fig. 6A).The heatmap showed the top ten genes up in the Pa + mAb group compared to BC+mAb group (Fig. 6B) .The results of KEGG enrichment analysis revealed that these DEGs were closely associated with some tumor-related pathways, including the PI3K/Akt signaling pathway, the calcium signaling pathway, the proteoglycans in cancer signaling pathway and the PPAR signaling pathway (Fig. 6C-D).

**Fig. 7:**
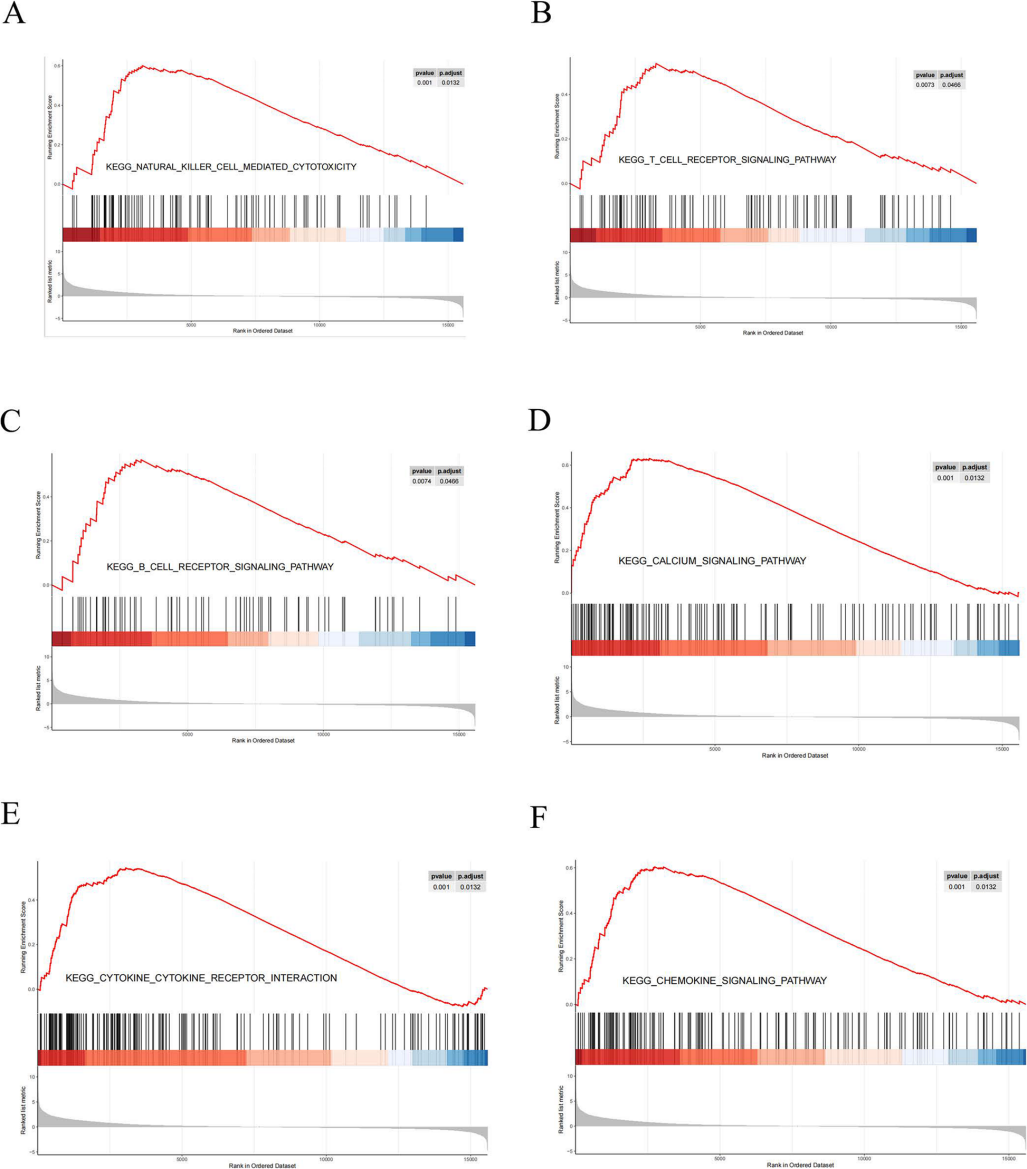
The GSEA analysis results of the up-regulated signaling pathways in Pa+mAb group mice. The graphs indicated significant up-regulation in the NK cell mediated cytotoxicity (**A**), T cell receptor signaling pathway (**B**),B cell receptor signaling pathway (**C**), calcium signaling pathway (**D**), cytokine_cytokine receptor interaction (**E**) and chemokine signaling pathway (**F**) in the Pa + mAb group

The results of GSEA demonstrated that the pathways associated with the antitumor immune response were remarkably upregulated in the Pa + mAb group, including “natural killer cell-mediated cytotoxicity”(*p*=0.001), “T cell receptor signaling pathway”(*p*=0.0073), “B cell receptor signaling pathway”(*p*=0.0074), “calcium signaling pathway”(*p*=0.001),“cytokine-cytokine receptor interaction”(*p*=0.001) and “chemokine signaling pathway”(*p*=0.001)(Fig. 7A-F).

## Discussion

Solid tumors frequently employed multiple drug resistance mechanisms to evade immune response, leading to a notably low overall response rate to anti-PD-1/PD-L1 immunotherapy, which has emerged as a pressing challenge in this field. Existing solutions to this issue encompassed the incorporation of chemotherapy, radiotherapy, angiogenesis inhibitors alongside anti-PD-1 immunotherapy, the application of dual immune checkpoint blockade or co-stimulatory molecule agonist in conjunction with anti-PD-1/PD-L1 drugs, the combination of targeted therapy (excluding angiogenesis inhibitors) with anti-PD-1/PD-L1 treatments and the innovation approach of FMT in combination with anti-PD-1/PD-L1 therapies [26]. Gut microbiota combined with anti-PD-1/PD-L1 immunotherapy was a novel and intriguing strategy but the precise mechanisms and optimal protocols of which remain unclear. Our study contributes fresh evidence regarding the efficacy, specific protocols, and underlying mechanism of this therapeutic approach, drawing insights from both animal models and clinical samples.

It has been reported that the administration of the probiotics *Lactobacillus rhamnosus* probio-M9 could enhance the therapeutic effect of anti-PD-1 immunotherapy, and significantly increase the abundance of *P. distasonis* [18]. A recent study further revealed that the relative abundance of *P. distasonis* and *Bacteroides vulgatus* was notably elevated in NSCLC patients who exhibited a more favorable therapeutic response to anti-PD-1 immunotherapy. Additionally, the administration of Ginseng polysaccharides was shown to restore the therapeutic efficacy in tumor-bearing mice who had received fecal material from patients with a poor response to immunotherapy, subsequently increasing the abundance of *P. distasonis* [19]. These findings collectively suggested a close association between *P. distasonis* and the host’s anti-tumor immune response, indicating that *P.distasonis* administration might enhance the effectiveness of anti-tumor immunotherapy.

*P. distasonis* had been substantiated to play a role in treating certain diseases by actively participating in metabolism and immune processes [27, 28]. In our research, 16S rDNA analysis revealed a significant decrease in the abundance of *P. distasonis* in patients with bladder cancer compared to healthy individuals. This reduction in abundance might contribute to the reduced effectiveness of ICIs in bladder cancer patients. Therefore, restoring the intestinal ecology in these patients could potentially enhance the efficacy of ICIs. To investigate this, we constructed an animal experiment model to examine T cell subset densities within the tumor microenvironment. We noted an increase in the densities of CD4^+^T and CD8^+^ T cells in the tumor of the Pa + mAb group, while the density of FoxP3^+^ cells showed no significant change. Our results indicated that combining *P. distasonis* with PD-1 inhibitors could improve immunogenicity by promoting the recruitment or expansion of CD4^+^T and CD8^+^T cells. This was consistent with previous findings suggesting that *P. distasonis* could support ICIs mediated anticancer immunity by stimulating of CD8^+^ T cell production [29, 30].

Concerning the biological mechanism, the DEGs analysis between the BC + mAb and Pa + mAb groups revealed enrichment in pathways related to tumor progression and antitumoral immune responses. These pathways encompassed calcium signaling, PPAR signaling, and proteoglycans in cancer. The role of calcium signaling in facilitating tumor cell proliferation, invasion, and interaction within the tumor microenvironment was well established [31]. Furthermore, BCG and its supernatant had been observed to stimulate bladder cancer cells to secrete pro-inflammatory cytokines such as IL-8 by activating calcium signaling [32]. The involvement of Peroxisome Proliferator-Activated Receptors (PPARs) in tumorigenesis and antitumor immune responses were also well noted [33]. For example, PPAR-γ activation could enhance PD-L1 expression in human cancer cells and organoids [34]. Agonists of the PGC-1/PPAR complexes had shown promise in increasing the proliferation and functionality of cytotoxic T lymphocytes, thereby improving the effectiveness of anti-PD-1 immunotherapy [35]. Moreover, Chondroitin Sulfate Proteoglycan 4 (CSPG4) exhibits increased expression in various cancers, including melanoma, breast cancer, and renal cell carcinoma, positioning it as a potential target for antitumor immunotherapy [36]. These insights collectively illuminated the complex biological mechanisms that might underlie the observed enhancement in antitumoral immune response in the Pa + mAb group through the enrichment of DEGs in these significant pathways.

In previous studies, various indicators had been proposed to predict the effectiveness of immunotherapy. PD-L1, recognized as a key and logical immune biomarker for PD-1/PD-L1 checkpoint blockade therapy, was notably linked with treatment outcomes in immune and tumor cells, as demonstrated in studies such as IMvigor 210 and Checkmate 275 [37, 38]. Beyond PD-L1, molecular and hematological markers had emerged as valuable predictors of therapy response and the prognosis of bladder cancer. Research showed that molecular subtypes serve as indicators for the effectiveness of cisplatin-based neoadjuvant chemotherapy in MIBC, with patients exhibiting a basal tumor type experiencing superior clinical outcomes [39]. Furthermore, recent findings suggested that Fibronectin-1 levels may forecast the success of immunotherapy in MIBC, with higher expression levels correlating with an increase in immunosuppressive cell infiltration [40]. Additionally, MDM2 had been identified as a marker linked with the anti-tumor immune response, offering prognostic insights for bladder cancer [41]. The presence of circulating tumor cells (CTCs) in the bloodstream also served as an indicator of progression and a poor prognosis for NMIBC and metastatic bladder cancer patients [42, 43].

Therefore, identifying predictive indicators for immunotherapy outcomes via gut microbiota analysis remained a task of considerable importance and challenge. By integrating evaluation and precise modulation of gut microorganisms, we could improve the accuracy of both predicting and augmenting immunotherapy's effectiveness for patients. The comprehensive strategy was promising for overcoming the intricate challenges involved in predicting treatment responses across different cancers. Our study underlined the innovative potential of combining *P. distasonis* with anti-PD-1 therapy to boost the efficacy of anti-PD-1 immunotherapy in treating bladder cancer. This enhancement likely results from the stimulation of pathways related to immunity and tumor suppression, providing crucial insights for future clinical approaches and mechanisms research.

Nevertheless, it was important to acknowledge the limitations of our study. Firstly, The the clinical samples obtained from both healthy individuals and bladder cancer patients were relative small, lacking a more extensive clinical cohort for support. And we should also expand the animal trails to comprehensively investigating the impact of *P. distasonis* on immunotherapy efficacy. Secondly, regarding the research on the mechanism by which *P. distasonis* enhanced the efficacy of immunotherapy, we had only verified that it could enhance immune cell infiltration in the tumor microenvironment and predicted downstream mechanism pathways through RNA sequencing, including calcium ion pathways, PPAR pathways, etc. However, more potential mechanism pathways required further exploration. Finally, while positive results were achieved in animal models, the safety and efficacy of *P. distasonis* for human bladder cancer patients still required further validation to assess their potential application. Therefore, in the future, we will make more efforts to address these shortcomings.

## Conclusion

Combining the delivery of *P. distasonis* with anti-PD-1 treatment represents a novel strategy aimed at augmenting the effectiveness of anti-PD-1 immunotherapy. This enhanced effect is likely achieved through the activation of immune and antitumor-related pathways.

### Supplementary Information

Supplementary Material 1: Supplement Figure 1. The relationship between the gut microbiota composition inbladder cancer patients with gender and smoking status. The α-diversity of Shannon index (A); Simpson index (B) and Observed species (C) results showed no gut microbiota difference in patients with gender. The α-diversity of Shannon index (E); Simpson index (F) and Observed species (G) results showed no gut microbiota difference inpatients with smoking status. The β-diversity of PCoA result (D and H) also showed no difference gut microbiota composition in bladder cancer patients with gender or smoking status.

## Data Availability

All data involved in this study has been submitted to the NCBI database, accession number PRJNA884863 and PRJNA884991. Additional information and requests for additional data and code should be directed to Peng Wu (doctorwupeng@gmail.com).
