# Supplementary figures and images for: Gut microbiota Parabacteroides distasonis enchances the efficacy of immunotherapy for bladder cancer by activating anti-tumor immune responses
Source: BMC Microbiol. 2024 Jul 3;24:237. doi: 10.1186/s12866-024-03372-8 (PMC11221038; doi:10.1186/s12866-024-03372-8)

A

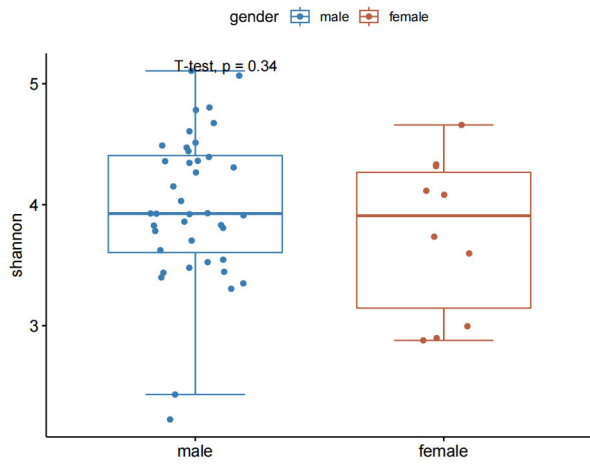

B

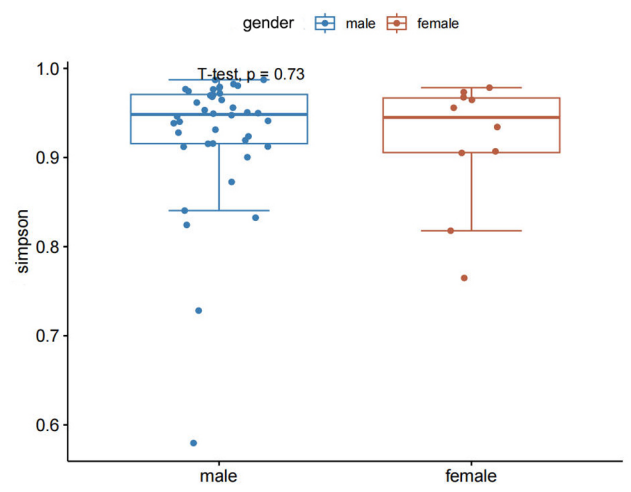

C

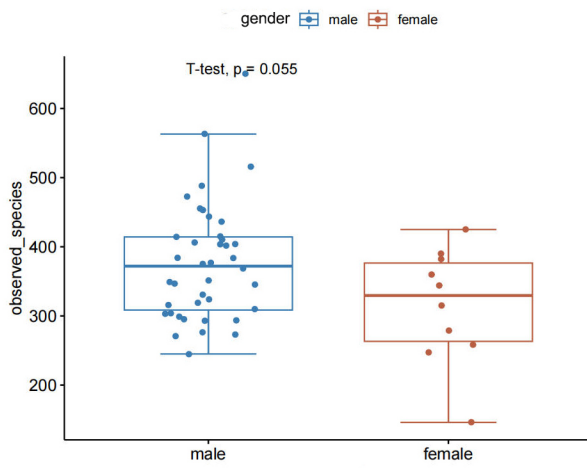

D

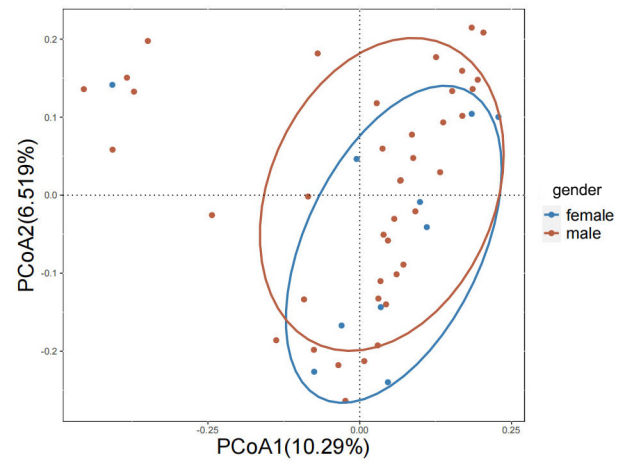

E

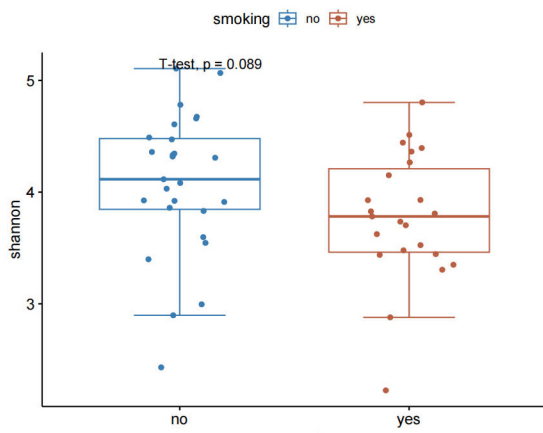

F

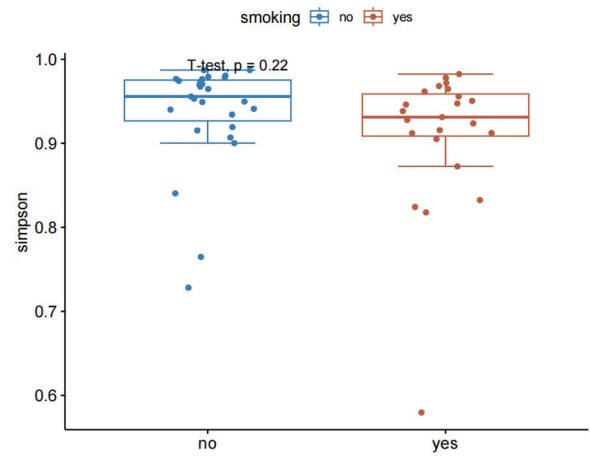

G

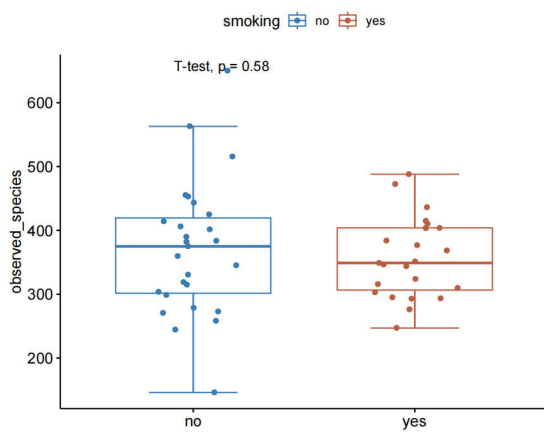

H

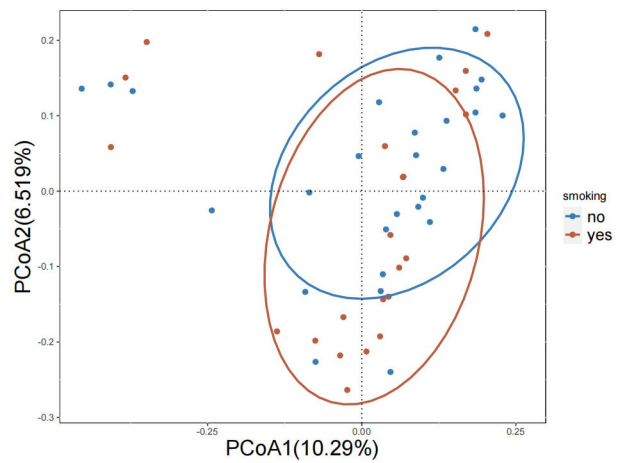

Supplement: Supplementary file 1 — Supplementary Material 1: Supplement Figure 1. The relationship between the gut microbiota composition inbladder cancer patients with gender and smoking status. The α-diversity of Shannon index (A); Simpson index (B) and Observed species (C) results showed no gut microbiota difference in patients with gender. The α-diversity of Shannon index (E); Simpson index (F) and Observed species (G) results showed no gut microbiota difference inpatients with smoking status. The β-diversity of PCoA result (D and H) also showed no difference gut microbiota composition in bladder cancer patients with gender or smoking status. [file 12866_2024_3372_MOESM1_ESM.pdf]
